# Peutz–Jeghers Syndrome in a Young Ethiopian Male: A Case Report

**DOI:** 10.1155/crgm/3667487

**Published:** 2025-05-07

**Authors:** Abate Bane Shewaye, Kaleb Assefa Berhane

**Affiliations:** ^1^Department of Internal Medicine, Adera Medical and Surgical Center, Addis Ababa, Ethiopia; ^2^Department of Internal Medicine, College of Health Sciences, Addis Ababa University, Addis Ababa, Ethiopia

**Keywords:** cancer risk, Ethiopia, gastrointestinal polyps, hamartomatous polyps, mucocutaneous pigmentation, Peutz–Jeghers syndrome

## Abstract

Peutz–Jeghers syndrome (PJS) is a rare autosomal dominant disorder characterized by hamartomatous polyps in the gastrointestinal (GI) tract, pigmented mucocutaneous lesions, and an increased risk of cancer. We report a case of a 22-year-old male from Ethiopia who presented with recurrent abdominal pain and a history of surgery for bowel obstruction. Endoscopic evaluation revealed multiple polyps in the stomach, ileum, and colon, which were confirmed histopathologically as hamartomatous polyps. Mucocutaneous pigmentation and family history of GI symptoms and maternal breast cancer led to the diagnosis of PJS, despite the unavailability of genetic testing. The patient underwent therapeutic polypectomy and was advised on cancer surveillance. This case highlights the importance of recognizing and managing PJS in resource-limited settings, emphasizing the need for early diagnosis and vigilant surveillance to prevent complications, especially when genetic testing may not be readily available.

## 1. Introduction

PJS is a rare autosomal dominant disorder characterized by GI polyposis, mucocutaneous pigmented macules, and an increased risk of cancer. The condition is caused by germline mutations in the Serine/Threonine Kinase 11/Liver Kinase B1 (STK11/LKB1) gene, a tumor suppressor involved in cell cycle regulation. Mutations in STK11 are present in 50%–80% of PJS cases, with the remainder likely due to de novo mutations [1, 2].

PJS occurs across all ethnic groups, affecting males and females equally, with an estimated incidence of 1 in 25,000 to 300,000 births [2]. Patients are at a significantly higher risk of developing GI cancers (colorectal, pancreatic, and gastric cancers) as well as non-GI cancers, including those of the breast, ovary, uterus, cervix, lung, and testicles [1, 3].

The two classic manifestations of PJS are mucocutaneous pigmented macules and hamartomatous polyps in the GI tract. The average age of diagnosis is 23 years. Pigmented macules, present in over 95% of patients, typically appear during childhood and are found on the lips, buccal mucosa, and other areas like the nose, eyes, genitals, and fingers [1, 2]. Hamartomatous polyps, which develop in the first decade of life, are most commonly located in the jejunum but can occur throughout the GI tract and at extraintestinal sites. These polyps may lead to complications such as intussusception, bowel obstruction, and chronic bleeding. Bowel obstruction due to intussusception is often the initial presentation of the disease. However, obstruction can also occur as a result of de novo colonic malignancy [2, 4].

Diagnosis is based on the presence of at least two of the following criteria: family history of PJS, mucocutaneous pigmentation, and GI hamartomatous polyps. Genetic testing plays a crucial role in confirming the diagnosis. A detailed clinical history, including information from childhood, and familial history is also essential. Family members of confirmed PJS patients should undergo genetic testing. However, it is important to note that not all families with PJS have mutations in the STK11/LKB1 gene, so a negative genetic test does not exclude the diagnosis [3, 5].

Management of PJS primarily involves surveillance and treating complications, such as endoscopic removal of polyps and regular cancer screenings. Early detection and prevention are vital for reducing the morbidity and mortality associated with this syndrome [1].

Here we report a case of PJS in a young Ethiopian male, highlighting diagnostic and management challenges in a resource-limited setting, the role of clinical diagnosis without genetic testing, and the need for awareness and surveillance to prevent malignancy.

## 2. Case Presentation

A 22-year-old male patient from Bechena, in the Amhara region, presented to Adera Medical and Surgical Center (AMSC), Addis Ababa, with a 3-month history of recurrent episodes of colicky abdominal cramping. He had visited a nearby medical center multiple times for this complaint and was prescribed unspecified medications, but experienced no improvement. He reported a history of bowel obstruction surgery 3 years ago and a recent right hemicolectomy 3 months prior to his presentation to our center for a similar presentation. His excisional tissue biopsy report showed a tubulovillous adenoma with low-grade dysplasia in the colon. He denied experiencing belching, vomiting, hematemesis, rectal bleeding, anemia-related symptoms, or weight loss. Additionally, the patient reported no history of smoking, alcohol use, diabetes, hypertension, or other chronic illnesses. He is an only child in his family and reported that his mother had similar GI symptoms before she passed away due to breast cancer 10 years ago.

**Figure 1:**
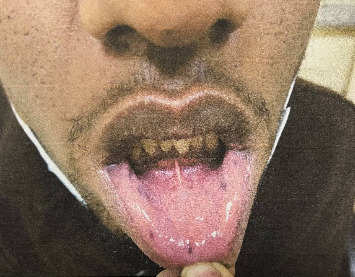
Hyperpigmented mucocutaneous lesions of the lower lip of the patient with PJS at AMSC, Addis Ababa, 2024.

His physical examination findings were largely unremarkable except for brownish macular spots on the lips (Figure 1). Abdominal examination revealed a healed surgical scar without tenderness, distension, or rigidity, and bowel sounds were active. No abnormalities were detected on rectal examination, and there was no evidence of rectal prolapse or blood. Additionally, there were no signs of gynecomastia, or testicular mass.

**Figure 2:**
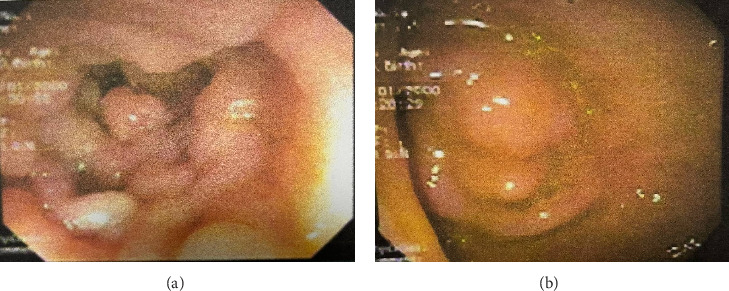
Endoscopic images of polyps in a patient with PJS at AMSC, Addis Ababa, 2024. (a) Polyps in the colon and (b) polyps in the gastric antrum.

Laboratory investigations, including complete blood count, liver and renal function tests, fecal occult blood test, carcinoembryonic antigen, and cancer antigens (CA-19–9 and CA-125), were all within normal limits. Abdominal computed tomography revealed polyposis in the stomach, ileum, and jejunum, along with evidence of the prior right hemicolectomy. Esophagogastroduodenoscopy identified multiple flat polyps in the gastric antrum, which was biopsied. Ileocolonoscopy showed numerous polyps in the colon and ileum (Figures 2(a) and 2(b)), and endoscopic polypectomy was performed. Histopathological analysis confirmed hamartomatous polyps with arborizing smooth muscle fibers with no evidence of atypia, dysplasia, or malignancy.

The patient was diagnosed with PJS based on the patient's clinical features, endoscopic findings as well as possible family history, even though genetic testing was unavailable. On his third month follow-up after his initial presentation to our center his abdominal cramping resolved and he had no subjective complaint.

## 3. Discussion

PJS was first referenced by Sir Jonathan Hutchinson in 1896 when he described circumoral pigmentation. However, it was in 1921 that the Dutch physician Peutz delineated the syndrome, which is characterized by mucocutaneous melanotic pigmentation and GI polyposis. Subsequently, Jeghers et al. provided an extensive overview of the syndrome in 1941 [6].

The first PJS case from Africa was reported in 1982, involving a 15-year-old female from Zimbabwe, who presented with colicky abdominal pain, vomiting, and fatigue, alongside circumoral pigmentation, anemia, and intestinal polyposis resulting in small bowel obstruction. The patient was treated with blood transfusion and conservative surgery [6]. Since then, fewer than 10 cases have been reported across the African continent. PJS is likely underdiagnosed in Africa due to limited awareness among healthcare providers and restricted access to diagnostic tools such as endoscopy, histopathological analysis, and genetic testing. Specific mutations of the STK11/LKB1 gene, may be more common in certain ethnic groups. For example, Zuo et al. reported two new mutations in three Chinese families that had not been previously described in White families, Zhao et al. found one novel mutation each in two Chinese patients, Chiang and Chen discovered five new mutations in eight unrelated Taiwanese families, and Jang et al. reported complete STK11 deletion in a Korean patient The absence of genetic studies in African populations also limits our understanding whether racial differences contribute to its lower reported incidence [5]. In Ethiopia, PJS was reported for the first time in 1994 by Mengesha et al. in a case report describing familial polyposis in two Ethiopians [7]. Here, we report the second case from Ethiopia. Unlike the first case, which appeared to result from a new genetic mutation, our patient has a family history suggestive of hereditary transmission.

Polyps associated with PJS typically manifest during adolescence and early adulthood, with one-third of affected individuals experiencing symptoms within the first 10 years of life. Polyps can develop anywhere in the GI tract, but the most common sites are the small bowel (78%), stomach (38%), colon (42%), and rectum (28%), as observed in this patient [4, 8]. The median age for the initial presentation of polyps is between 11 and 13 years, with approximately 50% of patients showing symptoms by age 20. The most common symptom is recurrent colicky abdominal pain due to obstruction and transient intussusception. Less frequent symptoms include melena and rectal bleeding, while hematemesis is rare [5]. Adult intussusception is rare, accounting for 1%–5% of intestinal obstructions and 5% of all intussusception cases. In PJS, it is the most common presentation, with up to 69% of patients experiencing intussusception during their lifetime. However, PJS is often not diagnosed until intussusception occurs [2, 9]. In our case, the patient underwent two surgeries for bowel obstruction caused by intussusception before being diagnosed with PJS. Increased awareness among clinicians is crucial, particularly in settings with limited healthcare access and cancer screening programs, highlighting the need for early surveillance and patient education.

The World Health Organization (WHO) diagnostic criteria for PJS involve a family history assessment. In cases with a positive family history, a diagnosis can be made with any number of histologically confirmed PJS polyps or the presence of characteristic mucocutaneous pigmentation. In the absence of a family history, the criteria require either three histologically confirmed PJS polyps or any number of polyps along with characteristic mucocutaneous pigmentation [10]. Although genetic testing for mutations in the STK11/LKB1 gene was not available, the patient in this case met the WHO diagnostic criteria for PJS due to the combination of classic clinical features of mucocutaneous pigmentation, and histologically confirmed polyps. Moreover, his recurrent episodes of abdominal pain, which required two surgical procedures for intestinal obstruction, along with his family history (maternal breast cancer and similar GI symptoms) further suggest the diagnosis of PJS.

Individuals with PJS face a lifetime cancer risk as high as 93%. The average age of cancer onset is 42 years, with colorectal cancer being the most prevalent (39% lifetime risk), followed by breast cancer in women (32%–54%) [1, 2]. People with PJS have a 15-fold increased risk of developing intestinal cancers compared to the general population [5]. Colonic malignancy at a young age can be the first presentation of the disease. In a case series, Pandit et al. reported an 18-year-old male patient who presented with signs of intestinal obstruction and underwent emergency surgery. The pathology report confirmed moderately differentiated adenocarcinoma of the colon with involvement of mesocolic lymph nodes [4]. Moreover, the risk of extra-intestinal cancers is elevated in PJS patients. Men with PJS are susceptible to Sertoli cell tumors of the testes, which can lead to hormonal disturbances such as gynecomastia and advanced bone age. Women with PJS are at increased risk for gynecological cancers, including benign ovarian sex cord tumors with annular tubules and mucinous tumors of the ovaries and fallopian tubes [1, 2].

Given the exponential cancer risk in PJS patients, treatment should focus on continuous monitoring and screening for malignancies. Several groups, including the National Comprehensive Cancer Network (NCCN), have proposed surveillance guidelines for cancer screening in PJS patients, largely based on expert opinions and limited observational data. The NCCN recommends yearly mammograms, breast MRIs, and clinical breast exams every six months starting at age 30 for breast cancer surveillance. Colonoscopy should be performed every 2–3 years beginning at age 18, and endoscopy is recommended every 2–3 years for gastric cancer screening from the same age. For small intestine surveillance, video capsule endoscopy or computed tomography/magnetic resonance imaging enterography is advised every 2–3 years starting at age 18. Pancreatic cancer screening, using endoscopic ultrasound or Magnetic Resonance Imaging/Magnetic Resonance Cholangiopancreatography, should begin at age 30–35 with yearly follow-ups. Cervical, uterine, and ovarian cancer screenings should commence at ages 18–20 with yearly pelvic exams and Pap smears [3].

Endoscopic resection of Peutz–Jeghers polyps can be performed to prevent the complications associated with polyp growth. Surgical intervention is required for polyps that cannot be managed endoscopically. In some cases, balloon-assisted endoscopy can enable resection of small intestine polyps, potentially avoiding the need for surgery. However, intussusception caused by large polyps usually necessitates surgical treatment, although endoscopic polyp removal following intussusception reduction via balloon-assisted endoscopy may be an option in certain cases [11]. Evidence linking hamartomatous polyps to malignancies supports the hamartoma-adenoma-carcinoma sequence hypothesis. However, the malignant potential of PJS polyps is still unclear, making it uncertain whether endoscopic polypectomy can help prevent or lower cancer risk [4]. In this patient, endoscopic polypectomies were done to reduce symptoms and prevent future complications, such as intussusception or bowel obstruction. He was counseled on the importance of regular surveillance and adhering to the screening schedules as per recommended.

## 4. Conclusion

PJS, though rare, should be considered in patients with recurrent colicky abdominal pain, particularly those with a history of bowel obstructions requiring surgical intervention. Key diagnostic clues include characteristic mucocutaneous pigmentation, GI polyposis, and positive family history, even in the absence of genetic confirmation. Increased clinical awareness, along with regular surveillance and timely management, is crucial in reducing morbidity and preventing malignancy in patients with PJS.

A key limitation of this case is the lack of genetic confirmation. Additionally, ensuring long-term follow-up to monitor surveillance adherence and cancer risk remains a challenge in resource-limited settings.

## Data Availability

The data that support the findings of this study are available from the corresponding author upon reasonable request.

## Nomenclature

AMSC: Adera Medical and Surgical Center
GI: Gastrointestinal
NCCN: National Comprehensive Cancer Network
PJS: Peutz–Jeghers Syndrome
STK11/LKB1: Serine/Threonine Kinase 11/ Liver Kinase B1
WHO: World Health Organization
